# Predicting the optimal timing for triggering in controlled ovarian stimulation: mature oocytes retrieval predictor

**DOI:** 10.1186/s12958-025-01489-7

**Published:** 2025-11-03

**Authors:** Masato Kobanawa

**Affiliations:** Kobanawa Clinic, 169-3 Tagiya, Omitama-shi, Ibarak-ken, Japan

**Keywords:** Assisted reproductive technology, Oocyte retrieval timing, Follicle-stimulating hormone, Controlled ovarian stimulation, Mature oocytes

## Abstract

**Background:**

The development of assisted reproductive technology (ART) has revolutionized infertility treatment; however, its success largely depends on effective controlled ovarian stimulation (COS) and the timing of oocyte retrieval. This study aimed to develop a regression equation model to optimize the timing of ovulation trigger in COS..

**Methods:**

We retrospectively analyzed 503 COS cycles (380 with follitropin alfa, 123 with follitropin delta) as training data. We modified the Follicle-To-Oocyte Index (FOI) and developed the Follicle-To-mature Oocyte Index (FmOI), which indicates how many mature oocytes (MII) were obtained for each antral follicle count. This index was used as an indicator for the retrieval of mature oocytes. When using FmOI as the objective variable, we selected relevant factors through Lasso regression analysis. Based on the obtained regression equations, the accuracy was compared and verified by predicting the number of MII in the test data.

**Results:**

Lasso regression analysis resulted in the creation of an FmOI prediction model using Initial serum FSH, number of follicles ≥ 14 mm, and total gonadotropin dose as explanatory variables. The regression equation model achieved Median Absolute Error values of 1.90 and 1.80 MII counts in the test data for the Alfa and Delta groups, respectively. Concordance index for MII prediction were 0.98 for follitropin alfa and 0.87 for follitropin delta. Use of the model showed higher CLBR in Alfa and non-inferiority in Delta than control group.

**Conclusion:**

This model reliably predicts the number of MII and optimizes trigger timing in COS. By considering key predictors, it provides a precise tool to enhance clinical outcomes in assistedreproductive technology .

## Introduction

Assisted reproductive technology (ART) has led to significant advances in the treatment of infertility and has fundamentally transformed reproductive medicine [1]. However, the overall success of ART relies heavily on optimizing controlled ovarian stimulation (COS) and precise timing of the oocyte retrieval procedure [2]. Effective COS is crucial to maximize the number of mature oocytes (MII) obtained, which is a major determinant of the success rate of ART [3].

Cumulative pregnancy and live birth rates per oocyte retrieval cycle have attracted worldwide attention as key performance indicators of ART [4, 5]. Important factors related to cumulative pregnancy and live birth rates include the number of oocytes and embryos available for transfer [6–9].

The introduction of recombinant follicle-stimulating hormone (FSH) formulations, such as follitropin alfa and delta, has provided more targeted stimulation protocols, allowing for personalized ovarian stimulation tailored to individual patient characteristics [10]. Various factors, including age, baseline antral follicle count (AFC), and basal serum hormone levels influence ovarian response, necessitating accurate predictive models to enhance treatment outcomes [11–20].

Indices have been developed to assess ovarian response, such as the Follicle-to-Oocyte Index (FOI), which is used to evaluate the efficiency of follicular development [21]. However, the ability to predict the number of mature oocytes (MII) that can be retrieved has not been fully addressed. We have, therefore, modified FOI to Follicle-to-mature Oocyte Index (FmOI) that quantifies the number of MII obtained in relation to the AFC, serving as a robust indicator to optimize the timing of oocyte retrieval. We aimed to use retrospective data from 380 cycles of follitropin alfa and 123 cycles of follitropin delta to establish a predictive model for accurately estimating FmOI and the number of MII, which may help in making more informed decisions regarding the optimal timing for triggering and enhance the clinical outcomes of ART.

## Materials and methods

Our study was approved by the Medical Corporation Kobanawa Clinic Ethic Screening Committee. After approval, this study was conducted with an opt-out disclosure of information with patients who provided informed consent.

This study consisted of two parts:

- Study 1: Development of the Mature Oocyte Retrieval Prediction Model.

It focused on the development of a predictive model to support optimal timing of trigger administration in COS , based on estimated FmOI and MII yield.

- Study 2: Validation of the Predictive Model in Clinical Practice.

It aimed to evaluate the model’s accuracy and clinical utility by retrospectively comparing outcomes from a prospectively managed cohort with data from previously treated patients.

### Study 1

Of the COS performed between April 2022 and December 2023, 380 cycles of follitropin alfa and 123 cycles of follitropin delta were retrospectively examined as training data. We included treatment-naïve women with COS who were treated with recombinant FSH monotherapy using either follitropin delta or follitropin alfa.

The required sample size was calculated based on Cohen’s effect size (f^2) for multiple regression analysis. Assuming a moderate effect size (f2 = 0.15), a significance level (α) of 0.05, and a statistical power of 0.8, with 9 explanatory variables (p) referred to later, the minimum sample size was estimated to be 120 using the following formula: n={(*p* + 1)+(Zα + Zβ)^2}/f^2 where Zα = 1.96 and Zβ = 0.84. This ensures sufficient power to detect significant predictors while avoiding overfitting [22].

Starting on days 1–3 of menstruation, patients were administered daily subcutaneous injections of follitropin alfa (Gonal F; Merck BioPharma, Tokyo, Japan) or follitropin delta (REKOVELLE; Ferring Pharma, Tokyo, Japan).

The initial dose of follitropin alfa (Alfa group) was determined using the gonadotropin starting dose calculator developed by Kobanawa with a fixed dose [23]. The daily individualized dose of follitropin delta (Delta group) was determined using the serum anti-Müllerian hormone (AMH) level within the previous 12 months and body weight, with a fixed dose used throughout the stimulation [24]. Gonadotropin-releasing hormone antagonists (Ganirest; Organon, Tokyo, Japan) were started at a dose of 0.25 mg/day when the primary follicle reached approximately 14 mm. When several leading follicles reached 17–20 mm, gonadotropins and GnRH antagonist doses of 0.25 mg/day were terminated, and on the same day or the next day, 250 µg of choriogonadotropin alfa (Ovitrelle/Ovidrel; Merck Biopharma, Tokyo, Japan) or 600 μg of GnRH agonist (Suprecur; CLINIGEN, Tokyo, Japan) was administered. Oocyte retrieval was performed 34–39 hours after triggering. Blood samples were collected during the study to assess AMH, FSH, luteinizing hormone (LH), estradiol, and progesterone levels. AMH concentrations were measured during screening before the start of the cycle and used to determine the starting dose of gonadotropins. AMH levels were measured using an automated Elecsys AMH assay (Roche Diagnostics, Basel, Switzerland). Serum samples were used to assess endocrine parameters (FSH, LH, estradiol, and progesterone). We considered oocyte retrieval at the time when the number of MII per AFC was the highest to be the most effective, and used a modified FOI (FmOI) as the objective variable for prediction [21].

After developing an FmOI prediction model using the training data, we validated the accuracy of 55 cycles using follitropin alfa and 37 cycles using follitropin delta with the test data. These 92 validation cycles (test groups) were retrospectively analyzed but were prospectively managed during January to March 2024, a period in which all COS cases were conducted using the prediction model we developed to support trigger timing decisions. These cases were not part of the model training and served as a real-world validation cohort. The accuracy was verified by comparing the difference between the actual number of MII when the FmOI was predicted and performed OPU at the precise time and the predicted number of MII calculated from the predicted FmOI and actual AFC. Median Absolute Error (MedAE), mean absolute error (MAE), and coefficient of determination (R2) were used as indicators of accuracy. For further validation, the bootstrap method was used for 10,000 simulations to predict the confidence interval of the estimated value [25].

### Study 2

For outcome comparison in Study 2, we retrospectively compared the test groups (Alfa and Delta), which were prospectively managed using in Study 1, with control groups consisting of COS cycles performed between January and December 2023 using either follitropin alfa or delta. All cycles were conducted under the same clinical conditions, including stimulation protocols, embryology laboratory environment, treating physicians, and embryo transfer protocols. Only patients who completed embryo transfer of all available embryos and reached live birth or cycle completion with no embryos remaining were included.

On each group, after oocyte retrieval, insemination or intracytoplasmic sperm injection (ICSI) were performed in training study. In insemination cases, MII status was inferred based on the presence of two pronuclei (2PN) approximately 18–20 h after insemination. In ICSI cases, cumulus cells were removed and MII status was directly assessed approximately 3–4 h after oocyte retrieval. Only oocytes with a visible first polar body were classified as MII and injected. MII was defined as oocyte confirmed by denudation for ICSI or oocytes confirmed zygotes with two pronuclei by insemination. Then the fertilized oocytes were cultured to blastocysts, which were all then frozen. Thawed embryos were transferred during the next menstrual cycle or later by hormone replacement cycles (HRC). In HRC, hormone replacement of estrogen (Estrana Tapes; Hisamitsu Pharmaceutical, Tokyo, Japan) at 0.72 mg ×4 every other day was started on days 1–3 of menstruation. With endometrial thickening confirmed to be at least 7 mm, progesterone (Utrogestan Vaginal Capsules; Fuji Pharma, Tokyo, Japan)200 mg ×3/day was commenced, and blastocyst transfer was performed six days later (*P* + 5). We performed a single embryo transfer (SET). Clinical pregnancy was defined as a case in which the fetal sac was confirmed by transvaginal ultrasonography within four to five weeks of gestation determined from the day of embryo transfer. After pregnancy, birth outcomes were tracked based on reports from the patients or the hospitals where the delivery occurred.

### Statistical analysis

Statistical analysis was performed using the chi-square test, t-test, Mann-Whitney U Test and Least Absolute Shrinkage and Selection Operator (LASSO) regression analysis [26] as applicable. Statistical significance was set at *p* < 0.05. Analyses were conducted using EZR (Saitama Medical Center, Jichi Medical University, Saitama, Japan), a graphical user interface for the R software (The R Foundation for Statistical Computing, Vienna, Austria) that enhances the R commander with additional biostatistical functions [27].

## Results

### Study 1

The patient characteristics in training data are presented in Table 1.

**Table 1:** Patient background data in training data are presented as mean ± standard deviation or median [min, Max]

Features	Alfa group *n* = 380	Delta group *n* = 123
Age (year)	35.35 ± 4.24	34.38 ± 4.10
AMH (ng/mL)	2.21 [0.02, 17.71]	2.89 [0.01, 9.00]
AFC (count)	8.00 [1.00, 50.00]	10.00 [1.00, 20.00]
Basal serum FSH (mIU/mL)	8.19 [0.54, 43.10]	7.00 [1.00, 21.00]
Trigger hour (hours)	37.32 ± 0.87	37.20 [34.00, 39.00]
Serum E2 on trigger day (pg/mL)	2568.95 [325.80, 12335.00]	3180.00 [282.00, 8286.00]
Serum P4 on trigger day (ng/mL)	1.05 [0.15, 10.92]	1.20 [0.10, 3.00]
Follicles ≥ 14 mm (follicles )	17.25 ± 9.77	14.68 ± 6.00
Body weight (kg)	56.00 [39.10, 106.20]	54.40 [39.00, 94.00]
starting dose (IU, µg)	225.00[100.00, 300.00]	8.33[6.00, 12.00]
Total dose (IU, µg)	2700.00 [600.00, 9000.00]	108.29 [54.00, 192.00]
Stimulation days (days)	12.00 [7.00, 22.00]	13.00 [8.00, 18.00]
Number of oocytes retrieved (oocytes)	12.00[1.00, 50.00]	12.00 [1.00, 24.00]
Number of mature (MII) oocytes (oocytes)	10.00 [1.00, 41.67]	11.00 [1.00, 24.00]
Trigger Method % (n)	hCG: 45.53(173), GnRH agonist: 54.47(207)	hCG: 72.36(89), GnRH agonist: 27.64(34)
FmOI	1.00[0.27,6.00]	1.27[0.14, 2.38]

The study population consisted predominantly of patients with normal to moderately decreased ovarian reserve. Specifically, those with polycystic ovarian morphology (PCOM, defined as AFC > 20) were limited to 3% (11/380) in the Alfa group and 0% (0/123) in the Delta group. Poor prognosis patients, defined as AMH < 1.2 ng/mL or AFC < 5, accounted for 16% (60/380) and 13% (16/123) in the Alfa and Delta groups, respectively.

In the Alfa group, the starting dose distribution demonstrated sufficient variability: 100 IU: 8% (30/380), 150 IU: 27% (104/380), 225 IU: 34% (128/380), 300 IU: 31% (118/380). This variability reflects individualized dosing based on patient characteristics such as AMH and age. Therefore, it supports the generalizability of the model across a wide clinical spectrum.

FmOI was used as an objective variable, while Age, AMH , basal serum FSH , trigger hour (hours from trigger induction to oocyte retrieval), serum E2 on trigger day , serum P4 on trigger day , number of preovulatory follicles ≥ 14 mm , total dose , and body weight were used as explanatory variables. The objective variable as FmOI was log-transformed to always be positive before use.

**Fig. 1:**
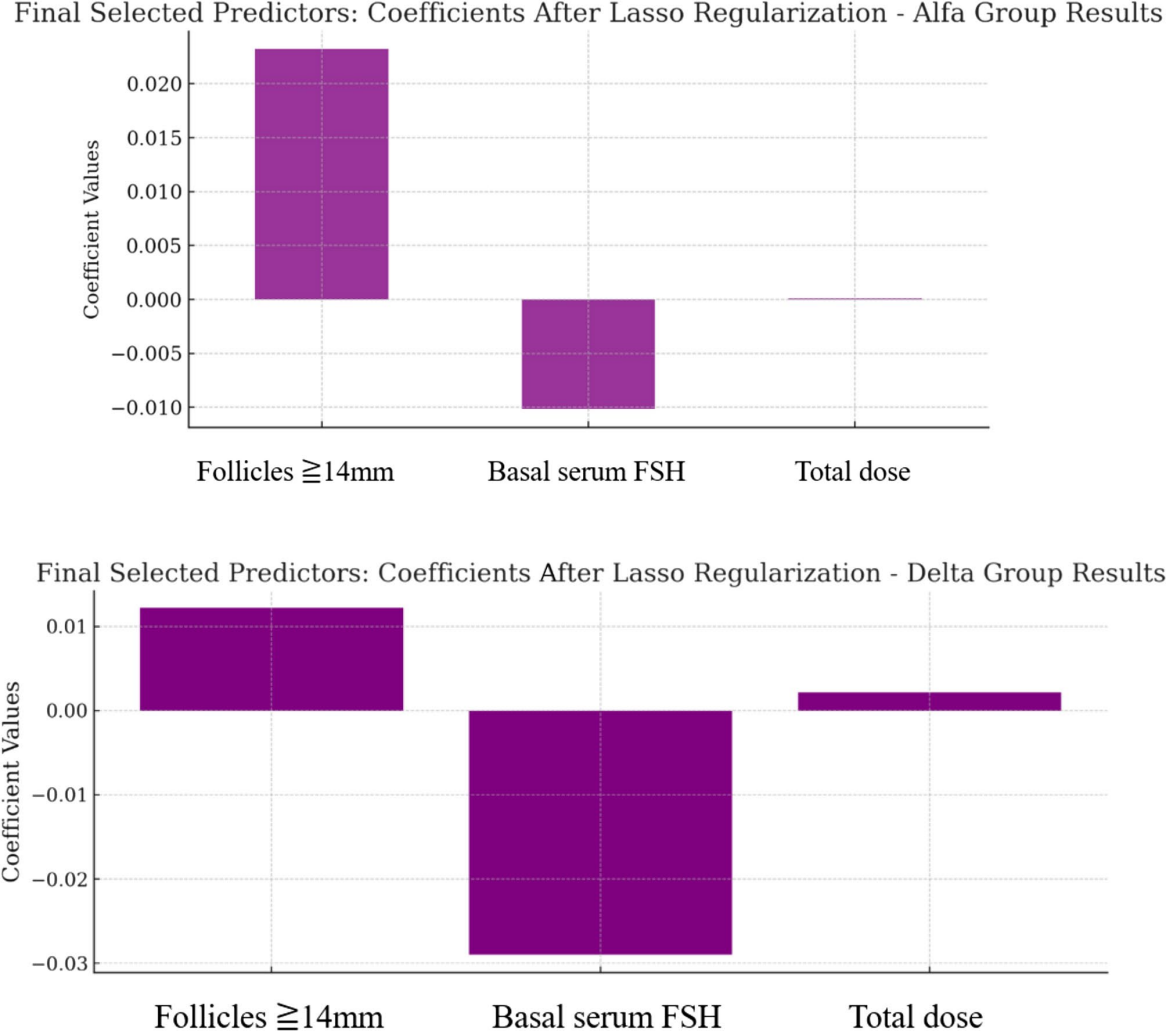
Lasso regression model predicting the natural logarithm of FmOI (log FmOI).The model was fitted with alpha = 0.075 (Alfa group) and 0.01 (Delta group) to identify key predictors of controlled ovarian stimulation. Three significant variables—basal FSH, total dose (IU), and the number of follicles >14 mm—were retained in the final model

We applied a LASSO regression model with alpha values of 0.075 (Alfa group) and 0.01 (Delta group). This model focused on selecting key predictors of COS. Among the analyzed variables, basal serum FSH level , number of preovulatory follicles ≥ 14 mm, and total gonadotropin dose emerged as significant contributors (Table 2; Fig. 1).

**Table 2:** The results of lasso regression analysis. FmOI prediction model use Basal serum FSH (IU/L), number of follicles ≥14mm, and total gonadotropin dose (IU or mcg) as explanatory variables

**Variable Alfa group**	**Estimate**	**95% CI Lower**	**95% CI Upper**	**Std. Error**	**t value**	***p* value**
(Intercept)	−0.36185	−0.59527	−0.12843	0.11871	−3.04819	0.002465
**Follicles ≥14mm (follicles)**	0.02321	0.017969	0.028451	0.002665	8.707933	9.92E-17
**Basal serum FSH (mIU/mL)**	−0.01016	−0.02369	0.003373	0.006882	−1.47609	0.140757
**Total** **dose** **(IU** **)**	0.000101	5.69E-05	0.000145	2.24E-05	4.505557	8.85E-06
**Variable Delta group**	**Estimate**	**95% CI Lower**	**95% CI Upper**	**Std. Error**	**t value**	**p value**
(Intercept)	−0.03075	−0.4878	0.426298	0.230822	−0.13323	0.894235
**Follicles ≥14mm (follicles)**	0.012174	−0.00116	0.025511	0.006735	1.80744	0.07322
**Basal serum FSH (mIU/mL)**	−0.029	−0.06244	0.004442	0.016889	−1.71709	0.088563
**Total dose (μg)**	0.002111	−0.00017	0.00439	0.001151	1.833913	0.069166

The basal serum FSH level reflected the baseline ovarian reserve and endocrine milieu prior to stimulation. Elevated or suppressed FSH levels can indicate diminished ovarian reserve or hypothalamic-pituitary axis dysfunction, respectively. As a continuous variable, basal FSH showed utility in stratifying patient response to stimulation and influencing follicular recruitment and the subsequent number of mature oocytes retrieved. Its selection in the LASSO regression underscored its centrality in predicting COS outcomes.

The number of follicles measuring ≥ 14 mm at the time of trigger administration served as a surrogate marker for ovarian responsiveness. This parameter directly correlated with the yield of mature oocytes and the likelihood of achieving favorable clinical outcomes. Its inclusion as a predictive factor highlighted its relevance in determining the efficacy of stimulation protocols and tailoring individualized COS strategies.

The total dose of gonadotropins administered during the stimulation phase represented a key modifiable factor in COS protocols. It reflects the ovarian sensitivity to exogenous stimulation and the clinician’s adjustments to achieve the desired follicular response.

Prediction models corresponding to COS with either follitropin alfa or follitropin delta were developed using following formulas:

### MII retrieval predictor (follitropin Alfa version)

$$\begin{aligned}\mathrm{Log}\left(\mathrm{FmOI}\right)\;&=\;0.02321039\;\ast\;\mathrm{follicles}\;\left(\geq14\;\mathrm{mm}\right)\\&+0.00010101\ast\mathrm{starting}\;\mathrm{dose}\ast\mathrm{stimulation}\;\mathrm{days}\;\\&-\;0.01015780\ast\mathrm{basal}\;\mathrm{serum}\;\mathrm{FSH}\;-\;0.36185141\end{aligned}$$

## MII retrieval predictor (follitropin delta version)

$$\begin{aligned}\mathrm{Log}\left(\mathrm{FmOI}\right)\;&=\;0.012174\ast\mathrm{follicles}\;\left(\geq14\mathrm{mm}\right)\;\\&+\;0.002111\ast\mathrm{starting}\;\mathrm{dose}\ast\mathrm{stimulation}\;\mathrm{days}\;\\&-\;0.029000\ast\mathrm{basal}\;\mathrm{serum}\;\mathrm{FSH}\;-\;0.030753\end{aligned}$$

When validated using each training dataset, the FmOI prediction accuracy showed an adjusted R^2^ = 0.125 and MedAE = 0.30 for the Alfa version, and adjusted R^2^ = 0.04 and MedAE = 0.30 for the Delta version. The accuracy of the predicted number of MII that can be expressed as FmOI×AFC using the predicted FmOI was adjusted R^2^ = 0.44 and MedAE = 2.34 for the Alfa version, and adjusted R^2^ = 0.25 and MedAE = 2.62 for the Delta version. The probability of the actual number of MII being less than the predicted number of MII was calculated using the bootstrap method for 1,0000 simulations as a prediction model. The Alfa and Delta versions were 54.2% and 56.0%, respectively, indicating that the predicted values tended to be higher than the observed values in most cases.

Because the MedAE of FmOI was 0.30 for both versions, we adjusted the model to predict the number of MII by (FmOI-0.3)×AFC, using the predicted FmOI-0.3 as the new predicted FmOI. The accuracy of the adjusted model was adjusted R^2^ = 0.45 and MedAE = 2.52 for the Alfa version and adjusted R^2^ = 0.04 and MedAE = 3.76 for the Delta version. Although the accuracy of the training data was slightly lower, the probability that the number of MII was less than the newly predicted number of MIIs was calculated using the bootstrap method in the same way, and the Alfa and Delta versions showed improvements of 24.5% and 12.0%, respectively.

following formulas:

### MII retrieval predictor (follitropin Alfa version)

$$\begin{aligned}\mathrm{Predicted}\;\mathrm{MII}\;=&\;\left[\exp\left\{0.02321039\ast\mathrm{follicles}\;\left(\geq14\;\mathrm{mm}\right)\;\right. \right. \\& \left. \left. +\;0.00010101\ast\mathrm{starting}\;\mathrm{dose}\ast\mathrm{stimulation}\;\mathrm{days}\;\right. \right. \\& \left. \left.-\;0.01015780\ast\mathrm{basal}\;\mathrm{serum}\;\mathrm{FSH}\;-\;0.36185141\right\}\;\right. \\& \left.-0.3\right]\ast\mathrm{AFC}\end{aligned}$$

### MII retrieval predictor (follitropin delta version)

$$\begin{aligned}\mathrm{Predicted}\;\mathrm{MII}\;=&\;\left[\exp\left\{0.012174\ast\mathrm{follicles}\;\left(\geq14\;\mathrm{mm}\right)\;\right. \right. \\& \left. \left. +\;0.002111\ast\mathrm{starting}\;\mathrm{dose}\ast\mathrm{stimulation}\;\mathrm{days}\;\right.\right. \\& \left. \left.-\;0.029000\ast\mathrm{basal}\;\mathrm{serum}\;\mathrm{FSH}\;-\;0.030753\right\}\;\right. \\& \left.-0.3\right]\ast\mathrm{AFC}\end{aligned}$$

**Fig. 2:**
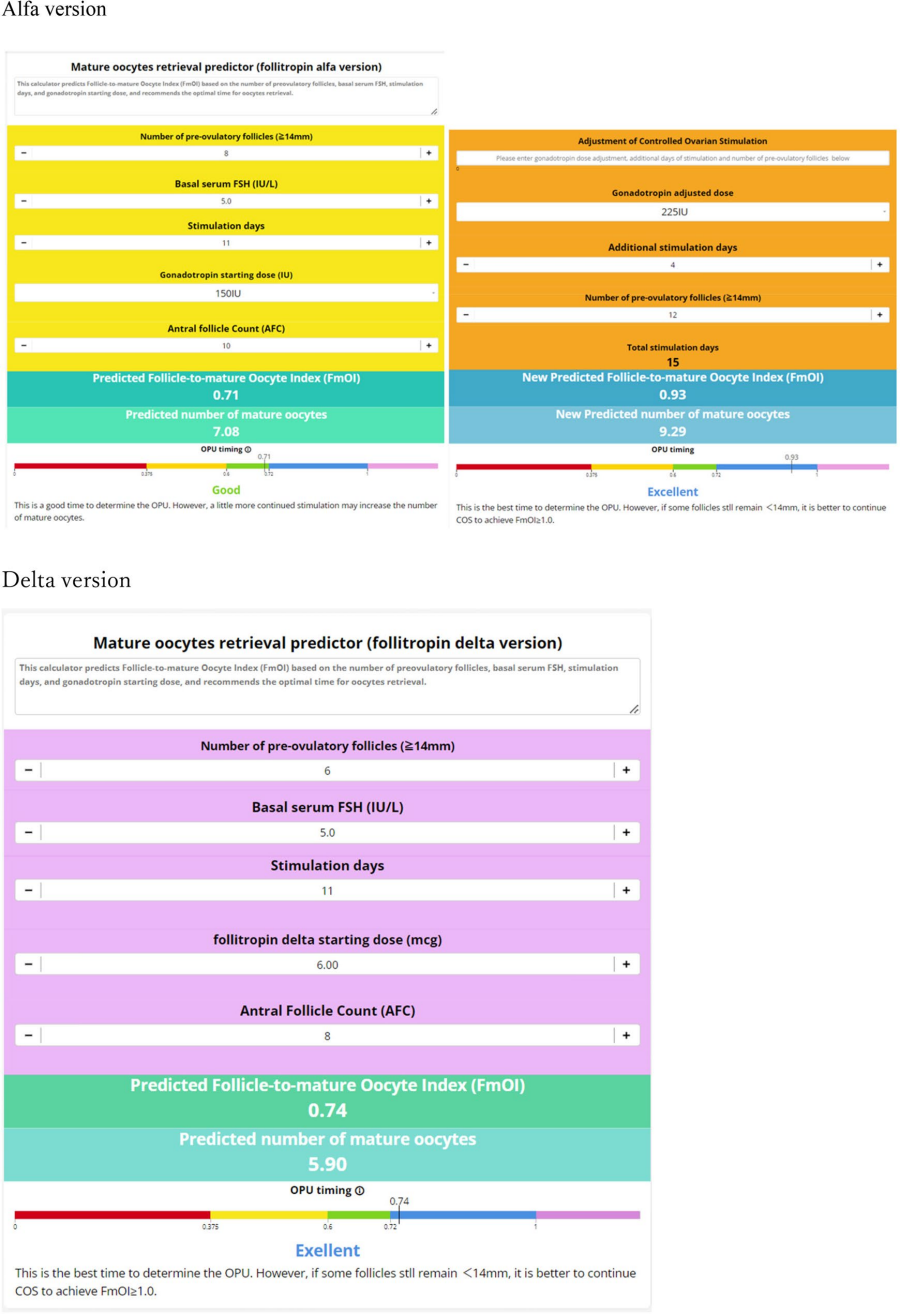
Prediction of optimal trigger timing and expected the number of mature oocyte (MII) retrieved.Basal serum FSH, total dose, and the number of follicles >14 mm were entered into the model to estimate the optimal timing for triggering in both Alfa and Delta models. In the Alfa model, dosage adjustment allowed reevaluation of the trigger timing. The predicted FmOI classified responses as good (0.6–0.72), excellent (>0.72), and perfect (>1.0). The expected number of MII was derived by multiplying AFC by the predicted FmOI

The MII retrieval predictor application, which uses this model, has been made available online as a tool [28] to assist in determining the timing of oocyte retrieval decisions or trigger induction by predicting FmOI. This can be accessed through the following links (Fig. 2):

Alfa version, https://www.calconic.com/calculator-widgets/mature-oocytes-retrieval-predictor-follitropin-alfa-version/6682b46762222a002ad642eb?layouts=true.

Delta version, https://www.calconic.com/calculator-widgets/mature-oocytes-retrieval-predictor-follitropin-delta-version/668bf8cd48abfd002abff688?layouts=true. 

The reference values for FOI, the source of FmOI, are minimum expected ≥ 50% and best practice goal ≥ 80%, and MII rate minimum expected ≥ 75% and best practice goal ≥ 90 according to the Italian Society of Fertility and Sterility and Reproductive Medicine (SIFES-MR) and the Italian Society of Embryology, Reproduction and Research (SIERR) [29]. According to the Maribor consensus, which provides key performance indicators for clinicians, the oocyte maturation rate is 75–90% [30]. From these values, we assumed that FmOI = FOI×maturation rate (molecular oocyte to mature oocytes). Good was defined as FmOI 0.6–0.72, excellent as FmOI >0.72, and perfect as FmOI >1, which is 100% mature oocytes recovery per AFC. The predicted number of mature oocytes (MII) was calculated by multiplying the input AFC with predicted FmOI.

The accuracy of the model was then tested using test data from patients for whom the trigger timing was guided by this model in actual clinical practice, with informed consent obtained. The test data included 55 patients with follitropin alfa and 37 with delta, all of whom met the same inclusion and exclusion criteria as the training cohort. Baseline characteristics of the test groups, along with those of the corresponding training groups, are presented in Table 3 for comparative purposes.

**Table 3:** Comparison of baseline characteristics between training and test groups (Alfa and Delta), presented as median [min, max]

Features	Test group(Alfa) *n* = 55	Training group(Alfa) *n* = 380	*p* value	Test group(Delta) *n* = 37	Training group(Delta) *n* = 123	*p* value
Age (year)	34.00 [24.00, 42.00]	35.00 [23.00, 42.00]	0.15973	34.00 [25.00, 42.00]	35.00 [24.00, 42.00]	0.58370
AMH (ng/mL)	2.69 [0.06, 19.88]	2.21 [0.02, 17.71]	0.16271	3.22 [0.19, 11.39]	2.89 [0.01, 9.00]	0.68272
AFC (count)	12.00 [2.00, 29.00]	8.00 [1.00, 50.00]	0.00002	11.00 [1.00, 25.00]	10.00 [1.00, 20.00]	0.07888
Basal serum FSH (mIU/mL)	6.32 [3.09, 15.50]	8.19 [0.54, 43.10]	0.00003	6.57 [1.86, 13.16]	7.00 [1.00, 21.00]	0.37111
Trigger hour (hours)	37.50 [36.72, 39.88]	37.32 ± 0.87	0.00039	37.50 [36.53, 39.08]	37.20 [34.00, 39.00]	0.02779
Serum E2 on trigger day (pg/mL)	3119.00 [383.10, 11623.00]	2568.95 [325.80, 12335.00]	0.03547	2780.00 [282.40, 14462.00]	3180.00 [282.00, 8286.00]	0.30877
Serum P4 on trigger day (ng/mL)	1.10 [0.25, 16.32]	1.05 [0.15, 10.92]	0.68330	1.10 [0.20, 1.41]	1.20 [0.10, 3.00]	0.77642
Follicles ≥ 14 mm (follicles)	14.00 [1.00, 37.00]	16.00 [1.00, 51.00]	0.12641	12.00 [1.00, 29.00]	14.68 ± 6.00	0.21328
Body weight (kg)	58.40 [43.90, 86.30]	56.00 [39.10, 106.20]	0.24784	56.00 [41.20, 116.20]	54.40 [39.00, 94.00]	0.17260
starting dose(IU, μg)	225.00 [100.00, 300.00]	225.00[100.00, 300.00]	0.03247	8.66 [6.00, 12.00]	8.33[6.00, 12.00]	0.69123
Total dose (IU, µg)	2550.00 [1050.00, 4800.00]	2700.00 [600.00, 9000.00]	0.11255	111.96 [21.00, 252.00]	108.29 [54.00, 192.00]	0.59713
Stimulation days (days)	14.00 [10.00, 22.00]	12.00 [7.00, 22.00]	< 0.00001	14.00 [10.00, 28.00]	13.00 [8.00, 18.00]	< 0.00001
Number of oocytes retrieved (oocytes)	13.00 [2.00, 31.00]	12.00[1.00, 50.00]	0.55965	10.00 [1.00, 24.00]	12.00 [1.00, 24.00]	0.08897
Number of mature (MII) oocytes (oocytes)	12.00 [2.00, 31.00]	10.00 [1.00, 41.67]	0.02002	9.00 [1.00, 22.00]	11.00 [1.00, 24.00]	0.18481
FmOI	1.06 [0.63, 2.50]	1.00[0.27,6.00]	0.21428	0.92 [0.12, 2.00]	1.27[0.14, 2.38]	< 0.00001

Each patient underwent COS using the same antagonist protocol as for the training data. One difference from the training data was the use of a predictive model for oocyte retrieval decisions and daily dose adjustments. The gonadotropin starting dose calculator developed by Kobanawa [23] was used as a guide to increase the dose when the response was poor or when the oocyte retrieval decision needed to be made earlier. No dose adjustment was made in COS using follitropin delta, and oocyte retrieval decisions were made by varying the duration of stimulation using this model.

**Fig. 3:**
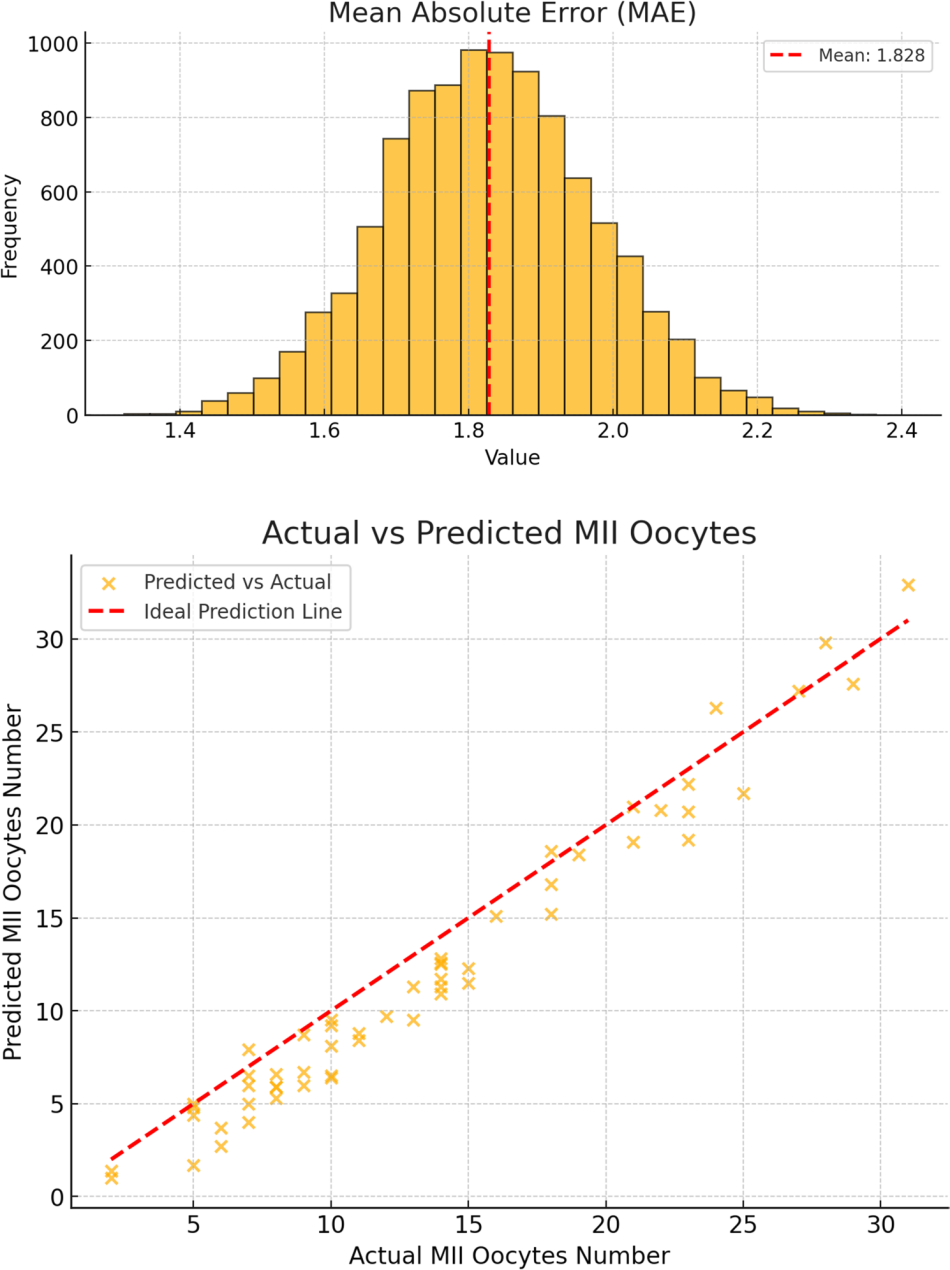
Bootstrap validation of the “Mature Oocytes Retrieval Predictor” (follitropin alfa version).This graph illustrates the model’s predictions for mature oocyte count, validated through 10,000 bootstrap simulations to evaluate reliability and accuracy

**Fig. 4:**
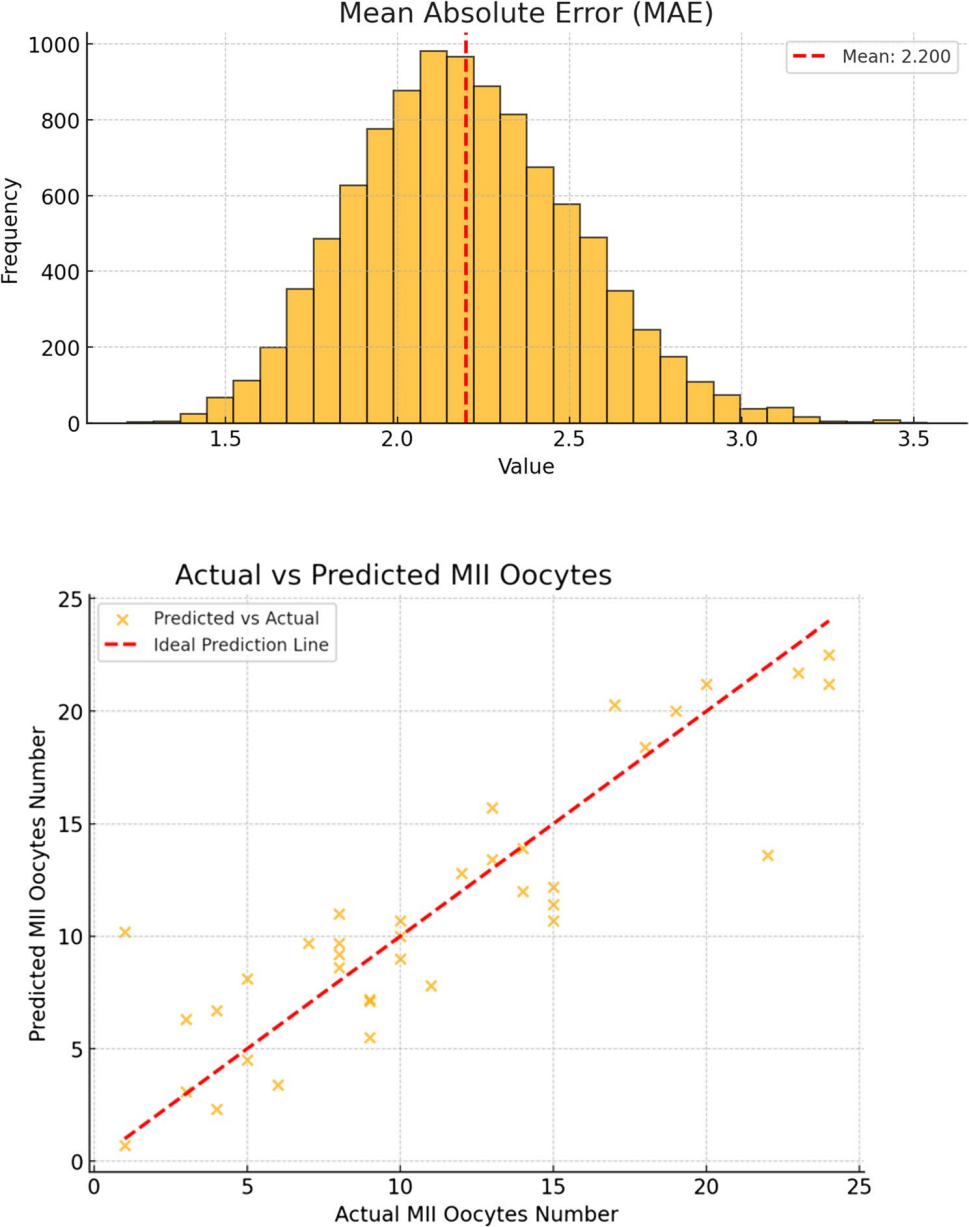
Bootstrap validation of the “Mature Oocytes Retrieval Predictor” (follitropin delta version).This graph shows the predicted number of mature oocytes generated by the model, validated through 10,000 bootstrap simulations to evaluate its reliability and accuracy

The results showed that MedAE of MII count was 1.90 and 1.80, MAE of MII count was 1.83 and 2.20, R2 was 0.91 and 0.79 for the Alfa and Delta groups, respectively. Validation by 10,000 simulations using the bootstrap method showed MAE = 1.83, 95% confidence interval (CI): 1.55–2.11 and C-index is 0.98 for the Alfa group (Fig. 3) and MAE = 2.20, 95% CI: 1.62–2.87 and C-index is 0.87 for the Delta group (Fig. 4).

#### Study 2

Previous data from OPU by physician perspectives were used as a control group to compare clinical performance with data using the predictive model. The Alfa model had significantly higher FmOI than the control group in terms of OPU performance. On the other hand, the Delta model showed no difference compared to the control group. Embryo culture results showed no difference in fertilization and blastocyst rates between the control group in either the Alfa or Delta models. Clinical outcomes also showed no difference in cumulative pregnancy rates, but cumulative live birth rates were significantly higher in the Alfa model than in the control group. On the other hand, the Delta model did not differ significantly from the control group. (Table 4)

**Table 4:** Patient background and clinical outcomes are presented as mean ± standard deviation or median [min, Max]

	Alfa Control *n* = 86	Alfa model *n* = 55	*P*-value	Delta Control *n* = 76	Delta model *n* = 37	*P*-value
**Age (yaers)**	35.00 [23.00, 42.00]	34.00 [24.00, 42.00]	0.3650	34.16 ± 3.83	34.00 ± 4.71	0.8599
**Body weight (kg)**	54.85 [40.50, 106.20]	58.40 [43.90, 86.30]	0.1471	53.90 [39.40, 82.40]	56.00 [41.20, 116.20]	0.1978
**AFC (follicles)**	14.50 [2.00, 30.00]	12.00 [2.00, 29.00]	0.37	15.00 [4.00, 30.00]	11.00 [1.00, 25.00]	0.0143
**AMH (ng/ml)**	2.52 [0.29, 17.71]	2.69 [0.06, 19.88]	0.6076	3.33 [0.68, 18.18]	3.22 [0.19, 11.39]	0.7090
**basal FSH (mIU/mL)**	7.68 [3.71, 15.21]	6.32 [3.09, 15.50]	0.0090	6.86 [2.88, 21.70]	6.57 [1.86, 13.16]	0.6999
**basal E2 (mIU/mL)**	29.98 [5.00, 98.79]	31.78 [6.97, 90.32]	0.6497	28.61 [5.00, 96.87]	29.51 [8.63, 59.00]	0.6586
**basal LH (mIU/mL)**	5.82 [0.40, 16.83]	5.85 [0.92, 19.30]	0.7178	5.88 [1.47, 13.75]	6.80 [1.83, 11.56]	0.1649
**stimulation days (days)**	12.00 [9.00, 22.00]	14.00 [10.00, 22.00]	0.0000	13.00 [10.00, 27.00]	14.00 [10.00, 28.00]	0.0000
**Starting dose ( IU**,**µg)**	225.00 [100.00, 300.00]	225.00 [100.00, 300.00]	0.2614	8.33 [6.00, 12.00]	8.66 [6.00, 12.00]	0.3638
**Total dose ( IU**,** µg)**	2700.00 [1000.00, 6600.00]	2550.00 [1050.00, 4800.00]	0.4484	108.29 [60.00, 226.62]	111.96 [21.00, 252.00]	0.6438
**trigger hour (hours)**	37.42 [36.33, 39.75]	37.50 [36.72, 39.88]	0.2983	37.21 [36.50, 38.75]	37.50 [36.53, 39.08]	0.0366
**Serum E2 on trigger day (pg/mL)**	3058.00 [479.20, 10278.00]	3119.00 [383.10, 11623.00]	0.7786	3394.50 [690.20, 8286.00]	2780.00 [282.40, 14462.00]	0.1853
**Serum P4 on trigger day (ng/mL)**	1.06 [0.22, 6.61]	1.10 [0.25, 16.32]	0.9192	1.30 ± 0.63	1.24 ± 0.77	0.6875
**Follicles ≥ 14 mm on trigger day (follicles)**	13.50 [2.00, 32.00]	14.00 [1.00, 37.00]	0.7493	14.00 [3.00, 32.00]	12.00 [1.00, 29.00]	0.1388
**Follicles ≥ 16 mm (follicles)**	11.00 [2.00, 29.00]	11.00 [1.00, 32.00]	0.8324	13.00 [3.00, 28.00]	11.00 [1.00, 29.00]	0.0986
**Follicles ≥ 18 mm (follicles)**	9.00 [1.00, 22.00]	9.00 [0.00, 27.00]	0.9358	9.00 [2.00, 27.00]	7.00 [0.00, 25.00]	0.0734
**Follicles ≥ 20 mm (follicles)**	4.00 [0.00, 16.00]	4.00 [0.00, 25.00]	0.6644	5.00 [0.00, 18.00]	5.00 [0.00, 18.00]	0.5550
** Number of oocytes retrieved(oocytes)**	14.50 [2.00, 32.00]	13.00 [2.00, 31.00]	0.6861	13.00 [1.00, 30.00]	10.00 [1.00, 24.00]	0.0283
** Number of mature (MII) oocytes(oocytes)**	13.50 [2.00, 32.00]	12.00 [2.00, 31.00]	0.987	13.00 [1.00, 30.00]	10.00 [1.00, 24.00]	0.0471
**FmOI**	0.90 [0.50, 6.00]	1.06 [0.63, 2.50]	0.0004	0.90 [0.14, 3.00]	0.92 [0.12, 2.00]	0.8709
**Fertilization methods (c-IVF) n(%)**	74 (86.0)	49 (92.7)	-	61 (80.3	33 (89.2)	-
**Fertilization methods (ICSI) n(%)**	12 (14.0)	4 (7.3)	0.4415	15 (19.7)	4 (10.8)	0.1511
**Number of 2PNs (zygotes)**	7.50 [0.00, 22.00]	7.00 [1.00, 22.00]	0.8471	8.50 [0.00, 23.00]	6.00 [0.00, 17.00]	0.1883
**Fertilization rate **	0.61 ± 0.21	0.62 ± 0.18	0.6678	0.64 [0.00, 1.00]	0.67 [0.00, 1.00]	0.5024
**Number of Good Quality Blastocysts (embryos)**	4.00 [0.00, 16.00]	5.00 [0.00, 17.00]	0.1072	4.50 [0.00, 18.00]	4.00 [0.00, 16.00]	0.9583
**Blastocyst rate **	0.52 [0.00, 1.00]	0.70 [0.00, 1.00]	0.0578	0.55 [0.00, 1.00]	0.67 [0.00, 1.00]	0.1989
**Number of embryo transfers **	1.00 [0.00, 5.00]	1.00 [0.00, 3.00]	0.1031	1.00 [0.00, 6.00]	1.00 [0.00, 3.00]	0.0836
**Cumulative PR n(%)**	63 (73.3)	47 (85.5)	0.1343	60 (78.9)	31 (83.8)	0.7217
**Cumulative LBR n(%)**	56 (65.9)	46 (83.6)	0.0346	44 (57.9)	24 (64.9)	0.6132

## Discussion

We developed a model that indirectly predicted the number of MII by predicting the FmOI. The purpose of the trigger is to induce final oocyte maturation so that the growing follicle yields an oocyte capable of fertilization and embryonic development [31]. This study was conducted to consider the possibility that more mature oocytes would increase the number of blastocysts after fertilization and ultimately increase the live birth rate because of the positive correlation that more mature oocytes would lead to increased 2PN zygotes, blastocysts, and live birth rates [32–34].

Several studies have been published regarding the use of machine learning and other techniques to predict the timing of COS. Canon used a linear regression model with follicle size and serum E2 as explanatory variables to predict the timing of COS triggering in patients. This predictive model, similar to ours, aimed to maximize the number of MII to standardize treatment decisions, thereby reducing the burden of medical decision-making on busy clinicians and increasing practice efficiency. Another potential use is to train clinicians to make clinical decisions by considering variables [35]. Letterie used regression analysis, random forests, support vector machines, logistic regression, and neural networks as algorithms with variables such as serum E2, follicle diameter, duration of stimulation days, and serum FSH level to develop a predictive model that could also be used for these applications [36]. Hariton performed regression analysis using a Light Gradient-Boosted Machine to show that optimizing the timing of trigger injection would significantly increase the number of 2PN zygotes and the total number of usable blastocysts resulting from an IVF stimulation cycle compared with the physician’s judgment [37]. Each prediction model included follicle diameter, follicle number, and gonadotropin levels, as in the present study, and the number and size of preovulatory follicles, which are also considered in actual clinical practice, are important predictors of oocyte retrieval decisions. Follicular diameter is an important guide for the acquisition of mature oocytes.

When deciding the trigger timing for oocyte retrieval, it has been reported that at least three follicles with a diameter of at least 17 mm [38] and several leading follicles reaching 16–22 mm are used as guideline [39]. Furthermore, oocytes retrieved from follicles < 12 mm in size are more likely to be GV oocytes, whereas those retrieved from follicles >17 mm in size are more likely to be mature oocytes [40, 41]. Additionally, mature oocytes can be obtained from follicle diameters of ≥ 16 mm, immaturity is more common in follicles < 14 mm [42, 43], and a recent review of trigger timing showed that the primary follicle diameter should be at least 14 mm [44]. Another study reported significantly lower maturation and blastocyst development rates when follicles < 12.5 mm in diameter were punctured [45]. We, therefore, set the follicle diameter to ≥ 14 mm in our predictive model.

Gonadotropin dosage is related to the daily dose and duration of stimulation and it affects the number of mature oocytes and clinical outcomes. Indeed, oocyte number increases in a dose-dependent manner with increasing FSH dosage [11, 12]. In patients with ovarian hyporesponsiveness, an increase in FSH dosage increases the number of oocytes retrieved by an average of 1–2, which may significantly reduce the cycle cancellation rate in cases of insufficient follicle growth [13]. Regarding the ESHRE guidelines of ovarian stimulation for IVF/ICSI, it is unclear whether high gonadotropin doses above a daily dose of 150 IU are recommended, and whether gonadotropin doses above 300 IU are not recommended for predictable poor responders. However, a higher number of oocytes and more embryos available for transfer at higher doses in poor-, normal-, and high-dose groups has been reported [46]. An increase in FSH dose has also been shown to reduce the duration of FSH administration [44]. Similarly, the duration of stimulation is negatively correlated with gonadotropin dosage, with higher dosages leading to shorter days and lower dosages leading to longer days [47, 48].

Thus, the duration of stimulation and gonadotropin daily dose may affect the acquisition of mature oocytes. However, there is a limit to the increase in the FSH dose [15, 16], and an increased dose does not necessarily contribute to an increased oocyte number [17]. Regardless of the number of oocytes retrieved and the age of the patient, the live birth rate may decreases as the total dose of FSH increases [18]. In a subgroup analysis of the same study, which was limited to patients with good prognosis, age < 35 years, BMI < 30, and no diagnosis of reduced ovarian reserve, endometriosis or ovulation disorders, showed similar results. This is because many cases of fresh embryo transfer were included, and it is possible that overstimulation with FSH elevated blood E2 and P4 levels, affecting endometrial decidualization [19, 20, 49]. Thus, administering excessive FSH doses is not an effective strategy because it has no positive effects on fresh embryo transfer or other procedures, and the cost is increased by overstimulation. Therefore, the FSH starting dose and stimulation days in COS are extremely important.

In addition, the present study used basal serum FSH values, which were not included in the parameters of previously reported trigger timing prediction models [50–52]. Serum FSH, AMH, and AFC levels are important factors to predict COS responsiveness. Especially for the antagonist protocol, baseline serum FSH levels are associated with the number of oocytes retrieved and the number of embryos [53]. More recently, it has been reported that basal FSH levels per cycle affect the number of oocytes retrieved in that cycle [54]. FSH receptor gene polymorphisms also promote an increase in serum FSH levels and decrease in FOI [21, 55, 56].

The *FSHR* rs6166 (c.2039 A >G, p.Asn680Ser) mutation is a prognostic indicator of the ovarian response to FSH stimulation and is associated with increased basal FSH levels, increased total gonadotropin requirements during ovarian stimulation, decreased peak estradiol levels, and a decrease in the number of oocytes retrieved [57–60]. In this case, a higher-than-usual dose of gonadotropin may be required for COS [57, 61, 62]. Conversely, the decreased sensitivity of FSHR may be overcome by increasing the administered FSH dose [60]. Against this background, we believe that the incorporation of the basal serum FSH level in the predictive model of oocyte retrieval decision is useful to adjust the total FSH dose and obtain an optimal number of mature oocytes in one COS.

Separate predictive models using the two FSH preparations were also developed in this study. FSH is a glycoprotein composed of two non-covalently linked polypeptide chains denoted by α and β, while follitropin delta has a glycan structure similar to endogenous FSH, containing not only α2.3 sialic acid but also N-acetylgalactosamine (GalNAc) and α2.6 sialic acid as glycan structure similar to endogenous FSH [63]. In contrast, follitropin alfa only has α2,3-linked sialic acid [64]. This difference in sialic acid content results in a lower clearance of follitropin delta from the serum, leading to greater exposure and pharmacodynamic response [63].

These differences in the sialic acid-binding isoforms influence follicular development and hormonal kinetics during COS. In the ESTHER study comparing follitropin delta and follitropin alfa, the total FSH dose tended to be lower and the duration of stimulation was longer in the follitropin delta group [65]. In the GRAPE study, which also compared follitropin delta and follitropin alfa, the total FSH dose was lower, and the duration of stimulation was significantly longer in the follitropin delta group [66]. In our previous study on the difference in follicle development dynamics between follitropin delta and alfa during COS, follitropin delta resulted in longer stimulation days and slower follicle development than alfa [67].

Predictive models for follitropin alfa can be used not only to search for trigger timing, but also to determine whether daily dose adjustment is necessary. Some studies have suggested that the starting dose is more important than the gonadotropin dose [68]. However, as mentioned, if the ovarian response is poor owing to FSH receptor gene polymorphisms, the FSH dose should be increased. In addition, if the starting dose is inappropriate, the threshold for follicle development (the FSH window), which is important for COS, may not be maintained [69–71]. The FSH threshold may be high, especially if the basal serum FSH level is high [72]. In such cases, gonadotropin dose adjustment may be necessary. A systematic review of the literature on gonadotropin dose adjustment in COS found dose adjustment in approximately 45% [73]. In our model of Alfa, the dose can be adjusted to enhance COS in consideration of FmOI values, and such a function may be useful for patients with difficult follicular development.

Our study has some limitations. The FmOI prediction model subtracted 0.3, the maximum FmOI deviation in the training data, from the predicted FmOI, to prevent an early oocyte retrieval decision owing to the low FmOI calculation. Therefore, the possibility that oocyte retrieval decisions may have been made too late in some cases cannot be ruled out. In addition, because the predictions of the FmOI and MII are calculated using regression equations, the accuracy of the predictions may decrease in patients whose AFC is too high.

Furthermore, a limitation of using 2PN as a proxy for MII in conventional IVF cycles is the potential exclusion of mature oocytes that failed to fertilize. However, 0PN embryos are generally associated with poor developmental outcomes [74], and their clinical use is not recommended according to the ESHRE guidelines [75]. Therefore, using 2PN-based MII confirmation aligns with the clinical objective of retrieving oocytes capable of producing viable embryos, which supports the relevance of our predictive model.

In clinical practice, this model may not only assist in identifying cycles with suboptimal response that require dose escalation but also serve as a tool to prevent overstimulation. When the predicted number of mature oocytes is excessive, especially in normal or high responders, clinicians may consider advancing the trigger timing to reduce the number of retrieved oocytes and avoid unnecessary risks and costs. Thus, the model offers flexibility to adjust COS to each patient’s profile, aiming not for maximum, but for optimal oocyte yield.

## Conclusions

The FmOI prediction model using the basal serum FSH level, number of follicles ≥ 14 mm, and total gonadotropin dose may optimize the timing of oocyte retrieval decisions and trigger induction to obtain the target number of MII in COS. Furthermore, adjusting the gonadotropin dose in cases of unexpected suboptimal response in COS using follitropin alfa may rescue optimal MII acquisition.

## Data Availability

The datasets supporting the findings of this study are available from the corresponding author upon reasonable request.

## Abbreviations

RT: Assisted Reproductive Technology
COS: Controlled Ovarian Stimulation
MII: Metaphase II
IVF: In Vitro Fertilization
AFC: Antral Follicle Count
FSH: Follicle-Stimulating Hormone
GnRH: Gonadotropin-Releasing Hormone
FmOI: Follicle-to-mature Oocyte Index
FOI: Follicle-to-Oocyte Index
AMH: Anti-Müllerian Hormone
LH: Luteinizing Hormone
E2: Estradiol
P4: Progesterone
ICSI: Intracytoplasmic Sperm Injection
MedAE: Median Absolute Error
R2: Coefficient of Determination
CI: Confidence Interval
2PN: Two Pronuclei
ESHRE: European Society of Human Reproduction and Embryology
SIFES-MR: Italian Society of Fertility and Sterility and Reproductive Medicine
SIERR: Italian Society of Embryology, Reproduction and Research
